# Emergency medicine training in the Netherlands, essential changes needed

**DOI:** 10.1186/1865-1380-6-19

**Published:** 2013-06-20

**Authors:** Menno I Gaakeer, Crispijn L van den Brand, Amanda Bracey, Joris M van Lieshout, Peter Patka

**Affiliations:** 1 Netherlands Society of Emergency Physicians, UMC Utrecht, Utrecht, The Netherlands; 2 Netherlands Society of Emergency Physicians, Medisch Centrum Haaglanden, The Hague, The Netherlands; 3 Gosford Hospital, Gosfor, NSW, Australia; 4 Emergency Medicine Department, Admiraal De Ruyter Hospital, Goes en Vlissingen, The Netherlands; 5 Emergency Medicine, Erasmus MC, Rotterdam, The Netherlands

**Keywords:** Emergency medicine, Training programme, Changes, The Netherlands

## Abstract

Since 2008, training for emergency physicians (EPs) in the Netherlands has been based on a national 3-year curriculum. However, it has become increasingly evident that it needs to expand beyond its initial foundations. The training period does not comply with European regulations of a minimum of 5 years. Adjusting to this European standard is a logical step. Experience with the 3-year Dutch training scheme has led to the general conclusion that this training period is too short. Recommendations for essential changes and the basis for their development are presented.

## Background

Since 2008, training for emergency physicians (EPs) in the Netherlands has been based on a national 3-year curriculum [1]. In November 2008, this training was also officially recognised by the Royal Dutch Medical Association (KNMG). To date, there are almost 300 trained and registered EPs working in 80% of the 96 Emergency Medicine Departments (EDs) in the Netherlands. It is estimated that over the next 5 years another 300 EPs will complete their training in one of the 28 recognised training hospitals. By then we expect all EDs will be working with EPs.

The first step in developing EM as a recognised medical speciality in the Netherlands was ensuring sufficient numbers of physicians trained in emergency medicine (EM) to staff all EDs. Since speciality training for EM was accepted by the KNMG, it has been supported by financial benefits from governmental training funds. This was important for further emancipation of emergency medicine training in the Netherlands [2]. In order to meet international training standards (EPs trained in the Netherlands are still not being recognised abroad), the next important step is further improving and extending emergency resident education [3]. It is becoming increasingly evident that it needs to expand beyond its initial foundations. The professional association ‘Netherlands Society of Emergency Physicians’ (NVSHA) has taken responsibility for monitoring and improving the form and quality of training of EPs in the Netherlands. It is taking the lead in bridging the gap between the current Dutch training programme and international training standards.

In this commentary, we evaluate the current 3-year EM training programme in the Netherlands. We identify the gaps in training and discuss possible improvements and the basis for their application in an upgraded training programme. The ultimate goal should be the efficient and intelligent professional future development of EM training in the Netherlands.

## Main text

### Current 3-year education of emergency trainees in the Netherlands

Currently, there are 28 training hospitals certified as individual training institutions, including six out of eight academic hospitals. More hospitals are currently considering applications for new additional training programmes.

There are obvious gaps in the current 3-year EP training and also, despite a national competency-based curriculum, there are significant local quality differences in training programmes on individual training sites.

The current education of emergency residents in the Netherlands lasts 3 years, but only 1 to 1½ years is actually spent in the ED. Residents spend the remainder of their time carrying out rotations in different medical specialty departments. Six medical specialties are mandatory: anaesthetics, intensive care, paediatrics, cardiology, general practice and a rotation with the Regional Ambulance Service. These disciplines are heavily involved in the training of emergency residents, but hardly offer enough specific educational needs for the emergency medicine residents. The duration of these rotations is not specified and goals are not uniformly assessed. Training hospitals may include additional speciality rotations in their local training programme. Except for an overseas rotation (which may only be undertaken during the last 6 months of training), the timing and sequence of rotations in the current 3-year training programme have not been set. The differences in the rotation, nature, duration, timing and goal assessment result in a wide variation between individual training schemes despite the existence of a national curriculum.

Although there is no international standard for the duration of residency training programmes, 3 years is considered to be too short a training period within Europe [4-6]. Secondary to both low-volume EDs in the Netherlands (15,000–50,000 patients/year) and excellent general practitioner (GP) care, there is insufficient exposure to seriously ill patients in a 3-year time frame. This lack of exposure is further intensified by the strict Dutch Labour Law, which limits working hours to 42 up to a maximum of 48 per week including education hours [7,8].

EM residents lack also their own role models. Secondary to an absence of EPs that have fulfilled the KNMG resident educator requirements (for example at least a 5-year post-training period), non-EM physicians were initially selected as EM resident educators. However, it is obvious that non-EM specialists don’t practice the specialty of EM in all its aspects and thus are suboptimally trained to educate EM residents.

There is no final board exam at the end of the training period, making it difficult to guard and ensure a national standard of training.

### Training programme from a European perspective

In 2006, European Union (EU) countries, together with other associated countries concerning the free exchange of persons and goods, attempted to develop minimum requirements for training for all medical specialities. These requirements were documented in a directive called the ‘Doctors’ Directive’ (EU Directive 2006/100/EC) [4]. Since this directive, EM (referred to as Accident and Emergency Medicine) has become one of the recognised specialities within the EU. This ‘Doctor’s Directive’ requires that any European country training scheme for emergency medicine must be a minimum of 5 years. In 15 of the 27 EU member states, EM is recognised as an independent medical speciality with a training scheme of 5 years or more. Nine of these 15 countries are listed in this ‘Doctors’ Directive’, which means that they met the requirements. The other six countries will be listed in the next revision of the Doctors’ Directive and by then will comply with the guidelines for EM training requirements. This means that the number of European countries that recognise EM as an independent medical speciality with a training programme of 5 years is growing. Furthermore, from a practical perspective there is an awareness of the urgent need for independent, well-educated EPs.

Following requests from the UEMS (European Union of Medical Specialists), a European curriculum for the education of EM residents has been established [5]. This European curriculum has been approved by both the EuSEM (European Society for EM) and the UEMS Multidisciplinary Joint Committee for EM. It encompasses surgeons, physicians, anaesthetists and paediatricians alongside EPs. To comply with the regulations of the EP curriculum, a minimum training time of 5 years was considered necessary. During this 5-year training scheme, a minimum of 3 years must be spent in an ED.

In October 2011, the UEMS established an EM section as this specialty now has an identical status to all other medical specialty disciplines within Europe.

### Considerations and recommendations regarding the training of emergency doctors

Still, some of the deficiencies identified above need to be addressed during the residency years. We suggest adjustments of the training programme and a basis leading to their implementation.

#### Adjusting the training time to the European standard of 5 years

The training period of Emergency Doctors in the Netherlands does not comply with the European regulations of a minimum of 5 years. Adjusting to this European standard is in itself a logical step. Also, experience with the Dutch training scheme has led to the general conclusion that a 3-year training period is too short. In order to address the multiple deficiencies mentioned above, significant additional training time to achieve core competencies is required.

#### Reducing the number of solitary training hospitals

Since 2008, 28 independent training hospitals have been established and certified in the Netherlands. This number is still increasing. Based on a needs analysis, the number of individual training positions in the Netherlands will be reduced over the next few years from 59 to less than 40 each yearly. From a training quality perspective, there is a need to correct the imbalance between the increasing number of training hospitals and fewer resident training positions available. Therefore, a reduction or integration of several training sites is inevitable. The financial and administrative interests that have made EM training appealing to many institutions has led to an unhealthy rigidity in the system that must now be overcome. Restructuring EM training from individual training sites to regional training programmes will reduce the number of independent training hospitals within the Netherlands. This should be the next logical step in the improvement of EM training.

#### Academic and non-academic united in one training programme

To further expand the area of EM, it is essential, in analogy to all other medical specialties, to acquire a higher academic (research and education) profile. By concentration and differentiation of acute care (such as occurs currently in academic hospitals), every emergency resident benefits by following part of the training in an academic hospital. Furthermore, the opposite holds true; emergency residents originally trained in an academic hospital would certainly also benefit from carrying out a part of their training in large non-academic hospitals that have more sizeable EDs to cope with increased volumes of patients. The cooperation of academic and non-academic hospitals within one regional training programme would ensure a more uniform national training structure, as already occurs in other medical speciality training programmes in the Netherlands.

#### More room for specific medical speciality placements

Medical specialty rotations are important to emergency resident training programmes. However their duration is often only 2 to 3 months. These short placements have many educational restrictions and are understandably related to a short overall training time of 3 years. Unfortunately, these short rotations offer the trainees insufficient opportunities to acquire adequate education to allow them to develop a sufficient level experience to practice EM independently. In other words, the time spent in medical specialty rotation must be followed for a sufficient period of time, and this requires the extension of the total duration of EM training. It is important to consider which medical specialty rotations should attain mandatory or priority standing. This should be in accordance with European regulations.

#### Emergency departments with emergency physicians present 24/7

An ED accredited for the training of emergency residents must be staffed with EPs 24 h per day, 7 days per week. Only then is it possible to provide immediate and direct supervision to emergency residents at all times. An additional advantage for the emergency residents is the ability to work with EP role models. Direct supervision also provides other important advantages, such as the safety of the learning environment and patient’s safety in the ED.

#### Emergency physicians as educators

Educators must be EPs who work in the ED and practice the speciality of EM in all its aspects. The old impression that a teacher and supervisor should be a medical specialist is not valid anymore. EPs who can fulfil the KNMG requirements (as applied to all medical specialty training programmes) to be emergency educators must take these positions. Continuous emancipation and qualification of emergency medicine educators remain important concerns.

#### Increased nationwide uniformity of the training programme

Since 2008, EM training has had a national curriculum in the Netherlands [1]. This curriculum describes the competencies that must be learned. However, translation of this in a uniform way to individual training hospitals still unfortunately results in a poorly defined training programme and an unsatisfactory profile for EPs. Defining skills and competencies only helps training hospitals to continue with their own programme instead of converging towards a common unified national training programme. We recommend developing a framework for training within which certain skills must be achieved, for example:

– clearly defined duration of medical specialty rotations,

– allocating medical specialty rotations to a specific training period,

– improving the quality of national teaching sessions and examinations.

#### Consequences of progress exams

Implementing a uniform national training programme makes it possible to connect consequences to the already established yearly national progress exam. Currently, this exam has no consequences connected to its results.

#### Ensuring new developments are addressed during training

Nationally and internationally, EPs are developing in areas such as emergency ultrasound, procedural sedation and analgesia, and regional anaesthetic blocks. These developments, as well as future developments, must be included within the training framework.

#### Selection of emergency residents

The area of EM is very popular for students and young doctors in the Netherlands. With limited available individual training places, it is time for the implementation of stricter selection criteria. For example, a minimum of 2 years of relevant clinical experience or post-graduate research period prior to commencement of the emergency training programme would be advisable. EPs must strive for a higher level of knowledge for themselves and for future generations. This is a challenge for both residents in training and established EPs.

#### Stricter criteria for training hospitals

Developing a new training scheme justifies the introduction of stricter requirements within the ED of training hospitals. As previously stated, a teaching ED must be staffed with EPs available 24/7 and not be dependent on unsupervised emergency residents. The educators must be EPs. The following criteria must also be considered for future accreditation of EM training departments:

– the ED must function as a 24/7 facility,

– emergency residents are given the opportunity to attend and manage all categories of patients,

– proper facilities available for the trainees within the ED,

– simulation training available in (or near) the ED,

– EPs should have well-developed and proven teaching skills, and

– emergency teachers and medical managers must all be EPs. An EP with a PhD should also be included in the training staff as the scientific co-ordinator.

## Discussion

The current EM-training programme was developed and officially accredited in the Netherlands in 2008. The period leading up to this accreditation by the KNMG was a unique situation, characterised by the evolution of a completely new system with a lot of new innovative elements in medical education. It was a unique situation where a new specific government-accredited emergency medicine training programme was developed based primarily on what was then politically feasible [9]. Unfortunately, it is not yet in agreement with European guidelines. Also the professional and scientific association (NVSHA) was and is convinced of the necessity of a national and European Union accredited training programme. However, a 3-year training programme was the only feasible compromise at that time because of overwhelming objection to the 5-year training programme, which was also proposed. Agreement of stakeholders with this limited training programme did however offer several advantages at the time, including the first formal recognition of the EP profession, assurance of training within the framework of the KNMG and training places financed with governmental training funds. It is obvious that the current training programme provides only a limited degree of experience in EM and in a manner that does not reflect international standards [4,5]. In relation to the fact that the area of EM has developed quickly over the last few years, this indicates that the training programme developed in 2008 is already insufficient to obtain satisfactory skills for practice in EM today.

In this period, a large number of EPs have successfully completed training and are now available to take care for the further development of improvement of the EM training programme. However, the reality is also that now, 5 years later, this training programme needs to be revised by implementing the aforementioned recommendations.

EPs are the first specialists within the Netherlands to qualify with a 3-year training programme. The question is whether the training of these professionals is possible in such a short training period. In our opinion, these suggested changes should be made in order to make the discipline of EP vital, sufficient and especially safe for the patient in acute need.

The argument for improved quality and patient safety in the ED is unabated. ED organisation must be further developed and improved. Questions about specific patient populations accessible to EPs, legal frameworks and increased patient expectations of a skilled medical workforce need to be addressed.

We are convinced that within 5 years EPs will be present 24/7 in every ED in the Netherlands. The presence of capable EPs will come through improved training and recognition that EM is indeed its own specialty area.

## Conclusions

EM in the Netherlands has developed from scratch to become an independent medical speciality, by no means a small achievement. Its development and liberation have brought with them new challenges for improvement of today’s EM training programme. The profession needs to make choices that will bring the training programme to a level that is connected to Europe as well as incorporated within the Dutch medical profession itself. This will in turn benefit patients admitted to the ED.

## Abbreviations

EP: Emergency physicians; KNMG: Royal Dutch Medical Association; ED: Emergency department; NVSHA: Netherlands Society of Emergency Physicians; EU: European Union; UEMS: European Union of Medical Specialists; EuSEM: European Society for Emergency Medicine.

## Competing interests

None of the authors have financial or non-financial competing interests.

Menno I. Gaakeer and Crispijn L. van den Brand are board members of the Netherlands Society of Emergency Physicians. Peter Patka is president of the Council of Training Directors of Emergency Medicine.

## Authors’ contributions

All authors contributed to the work presented in this article. MIG, the first author, initiated the manuscript and carried out the critical appraisal, data extraction, writing of the manuscript, translation and editing. CLvdB, the second author, carried out critical appraisal, data extraction and writing of the manuscript. AB, the third author, participated in writing the manuscript and translation. JML, the fourth author, carried out critical appraisal and participated in writing the manuscript. PP, the fifth author, was the primary supervisor, coordinated and supervised the discussion, manuscript writing and editing. All authors read and approved the final manuscript.

## References

[B1] Curriculum Opleiding tot Spoedeisende Hulp Arts http://www.nvsha.nl/index.php/opleiding-nascholing/opleiding/curriculum-en-formulieren

[B2] College Geneeskundig Specialismen, Besluit Spoedeisende Geneeskunde 09-01-2013 http://knmg.artsennet.nl/Opleiding-en-Registratie/RGS-1/Opleiding-spoedeisende-geneeskunde.htm

[B3] Holmes JL Emergency medicine in the Netherlands Emerg Med Australas 2010 6 75 81 10.1111/j.1742-6723.2009.01259.x 20152006

[B4] Council Directive 2006/100/EC 2006/100/EC http://www.eusem.org/cms/assets/1/pdf/directive2006%20100%20ce.pdf

[B5] Petrino R European Curriculum for Emergency Medicinehttp://www.eusem.org/curriculumofem.asp

[B6] IFEM Model Curriculum for Emergency Medicine Specialists http://www.ifem.cc/Resources/IFEM_Curricula_for_Emergency_Medicine.aspx

[B7] CAO ziekenhuizen 2011–2014 http://www.nvz-ziekenhuizen.nl/cao-kenniscentrum/cao/cao-ziekenhuizen-2011-2014

[B8] CAO UMC 2011–2013 http://www.nfu.nl/umcmedewerkers/cao/

[B9] Gaakeer MI van den Brand CL Peter P Emergency medicine in the Netherlands: a short history provides a solid basis for future challenges Eur J Emerg Med 2012 6 3 131 135 10.1097/MEJ.0b013e3283509d94 22293304

